# The Physics of Magnetic Resonance Imaging

**Published:** 1988-05

**Authors:** Paul Goddard, Peter Jackson


### The Physics of Magnetic Resonance Imaging: A Simplified Approach

**P Goddard. P Jackson.**

Physics is not a subject that endears itself to most people. It is, however, helpful to understand a background to the subject of magnetic resonance.

The pnenomenon of magnetic resonance is a natural property of matter that was discovered by American physicists in the 1940s. Magnetic Resonance Imaging is a development of this property in order to demonstrate the internal structures of the human body.

**Bar magnets**

Some nuclei of atoms possess magnetic moment and thus act like minute bar magnets. The most abundant atom in the human body to act in this way is that of hydrogen. Since the body consists mainly of water, which contains hydrogen nuclei, the information derived is principally concerned with the distribution of water.

The magnetic moment is present because the protons in the nuclei of atoms have a positive charge and also have spin. With charge and movement they will also act as small magnets. Paired protons will, however, spin in opposite directions thus cancelling the magnetic moments. Unpaired protons will result in a magnetic moment for the particular atom and the hydrogen atom is an obvious example consisting as it does of one proton and one electron.

**Water and fat**

Human beings are very largely made up of water and fat with hydrogen being bound in both. It is this hydrogen that is imaged using an MRI scanner.

**Scanning patients**

The patient is placed in a strong magnetic field. The hydrogen "bar magnets" will align either in the direction of the field or against it, with a small excess parallel to the main field.

The force of the magnetic field makes the spinning protons wobble around their axis like a spinning top. This movement is known as precession—an angled rotational motion.

A radio-frequency wave is introduced across the patient using transmission coils. If the frequencies are correct the protons of hydrogen will alter their angle of precession and will precess in phase with adjacent protons. The new angle is known as the "flip angle".

It is only when the frequency of spin and the frequency of the radio wave are effectively matching or proportional that the imparting of energy will occur. The process that occurs when the frequencies are appropriate in this way is known as "magnetic resonance".

The radio-frequency generator is switched off. At this time the protons are spinning together in the new angle. A coil placed across the line of movement will detect the generated radiofrequency wave. Since the transmitting generator has been switched off the protons move back to equilibrium either aligned or against the field. As they move back they move out of synchrony and the radiofrequency decays.

The amplitude of signal is related to the number of protons present and to a number of magnetic and physical properties of the matter being imaged. The total number of protons present for a given volume is known as the "proton density".

The length of time taken for decay of signal is known as the relaxation time. Two main relaxation factors are described—T1 and T2.

T1 is relaxation between the spinning proton and the environment. It is also known as spin–lattice relaxation.

T2 is relaxation between the different protons—the interference between neighbouring spinning protons. This is also known as spin–spin relaxation time and transverse relaxation time. The latter term is no longer a popular name since both factors operate in all 3 planes although T1 is predominantly in the longitudinal plane and T2 in the transverse plane.

Other important factors when imaging tissues are:

Chemical shift: the hydrogen in water and the in fat are differently bound and thus have slightly different resonant frequencies. This can result in an image where the fat and water does not completely overlap. This spatial difference is known as chemical shift.

Flow: substances such as water and blood flowing into and out of the scan will be interpreted as having low or no signal or as being in an inappropriate place on the image since the magnetised protons will not remain in their "correct" position long enough to give a signal at that site.

Susceptibility: this is essentially a measure of the magnetisability of the material. This is particularly important with metals such as soft iron—the high susceptibility will bend the lines of force of the magnetic field (remember iron fillings and magnets?) and create a signal void (lack of signal and thus a black area) on the picture.

**Gradients**

Magnetic gradients are superimposed on the main magnetic field in order to provide a means of spatially coding the signals received from the excited protons. This allows the site of emanation of the RF signal to be pinpointed since neighbouring points have different resonance frequencies.

**Creating Images**

The images are thus created by detecting the emanating signal, assigning a grey scale depending on the amplitude and the position in the picture depending on the radiofrequency.

The contrast between different tissues depends on the proton density and the relative balance of the different magnetic properties.

